# Empowering workplace allies for lesbian, gay, bisexual, and transgender employees to prevent and minimize psychological distress: A scoping review

**DOI:** 10.1002/cesm.12018

**Published:** 2023-06-23

**Authors:** Laurie Long Kwan Ho, Ankie Tan Cheung, Carlo Chak Yiu Chan, Eliza Lai Yi Wong, Wilson Wai San Tam, Wai Tong Chien

**Affiliations:** ^1^ The Nethersole School of Nursing, Faculty of Medicine The Chinese University of Hong Kong Hong Kong Hong Kong SAR; ^2^ Department of Urban Studies and Planning University of Sheffield Sheffield UK; ^3^ The Jockey Club School of Public Health and Primary Care, Faculty of Medicine The Chinese University of Hong Kong Hong Kong Hong Kong SAR; ^4^ Alice Lee Centre for Nursing Studies, Yong Loo Lin School of Medicine National University of Singapore Singapore Singapore

**Keywords:** Allyship, anxiety, depression, LGBT, stress, workplace

## Abstract

**Introduction:**

Lesbian, gay, bisexual, and transgender (LGBT) employees have increasingly reported experiencing different forms of workplace discrimination/harassment. Workplace allyship may be positively associated with psychological health through creating inclusive organizational cultures or reducing discrimination/harassment. However, comprehensive literature reviews or evidence syntheses on the effects of workplace allyship in mental health protection/promotion for LGBT employees are limited.

**Methods:**

This scoping review aimed to summarize available evidence regarding the effectiveness of workplace allies for LGBT employees in preventing/minimizing psychological distress and clarify the therapeutic components. This review included published research articles and grey literature identified through 11 electronic databases, a secondary search, and other sources.

**Results:**

We identified 27 relevant articles. Most included studies used cross‐sectional or qualitative research designs, and evidence from countries beyond the United States was limited. Three essential/effective components of workplace allies/allyship were identified that could create supportive/safe workplace relationships/climates: (a) knowledge, (b) empathy, and (c) action.

**Conclusions:**

Further longitudinal studies and controlled trials are needed to increase the quality of evidence on the effects and change processes induced by workplace allyship. Qualitative studies are also recommended to understand the health needs and mechanism of actions of workplace allyships in different LGBT communities.

## 1 INTRODUCTION

Many lesbian, gay, bisexual, and transgender (LGBT) employees have reported experiencing different forms of discrimination and harassment in the workplace because of their sexual orientation and/or gender identity [1, 2]. A population‐based survey of 5375 LGBT people in the United Kingdom reported that approximately one in six LGBT and one in three transgender employees experienced derogatory remarks (e.g., sexual harassment or offensive comments) and exclusion by coworkers [1]. Another survey involving 935 LGBT individuals in the United States found that more than 45% reported experiencing unfair treatment at work, including not being hired, being fired, or being harassed [3]. These findings suggested that a substantial proportion of LGBT employees face discrimination, stereotypic rejection, and even bullying or harassment at work, despite various governments (e.g., the United Kingdom and Canada) and companies across the world enhancing efforts to ensure workplace equality and inclusiveness.

Negative workplace experiences among LGBT employees are associated with psychological distress, including depression and anxiety, which have negative impacts on job functioning and life satisfaction [4]. Recent research suggested LGBT people have about a 2.5 times higher risk for depression and anxiety than their heterosexual and cisgender counterparts [5]. To avoid workplace distress caused by discrimination and harassment, up to half (26%–50%) of LGBT employees conceal their LGBT status from their supervisors and coworkers [3]. This may be attributable to the fact that LGBT employees who have “come out” (i.e., disclosed their sexual orientation or gender identity) at work are five times more likely to be fired or not be hired than those who have not come out [3]. Even if LGBT individuals conceal their sexual orientation or gender identity, extra energy and effort are needed to sustain this in the workplace, which could further increase psychological distress and reduce work satisfaction and commitment [6].

A recent conservative estimate indicated that LGBT individuals accounted for 6.8% and 4.5% of the United Kingdom and United States populations, respectively, although LGBT identities may not be visible [7, 8]. Despite the increasing number of LGBT people globally, there is little robust data estimating their total number in the general population or at different workplaces [8]. More attention is required to address or prevent mental health problems experienced by the LGBT community, especially those pertinent to the workplace, especially as work constitutes no less than one‐third of adult life.

Workplace allies are defined as individuals who are committed to preventing oppression by supporting and advocating for minority groups in the workplace [9]. LGBT workplace allies are individuals who support and advocate on behalf of LGBT employees to promote positive changes, combat oppression, provide emotional and social support, and enact changes in other employees' attitudes and behaviors, such as misconceptions and stereotypes toward LGBT individuals [10]. For LGBT employees, workplace allyship has been positively associated with psychological health through creating inclusive organizational cultures and reducing discrimination and harassment [11]. However, literature reviews on the effectiveness of this intervention approach and relevant guidelines for mental health protection and promotion among LGBT employees are limited. Existing reviews focused primarily on the workplace context without exploring/examining it in relation to the concept of allyship [12, 13]. This dearth of knowledge highlights the need to explore or identify available evidence for the most effective approach to empowering workplace allies for LGBT employees to prevent or minimize their psychological distress (i.e., depression, anxiety, stress), and clarify the essential or therapeutic components (i.e., active ingredients of an effective intervention) of allyship.

## 2 METHODS

### 2.1 Aims

The research questions addressed in this review were: (1) “What are the effects of workplace allies of LGBT employees on preventing or minimizing their psychological distress (i.e., depression, anxiety, stress) in different workplaces and cultural contexts?,” and (2) “What are the most essential or effective components of workplace allyship?” This review aimed to (1) summarize available studies and associated evidence regarding the effects of workplace allies for LGBT employees in preventing/minimizing psychological distress or improving/changing the working environment/relationships, and (2) identify essential or effective components/ingredients of workplace allyships.

### 2.2 Design

We conducted a scoping review to identify published and grey literature that evaluated allyship interventions for the LGBT community. In addition, this evidence synthesis approach could address an exploratory research question that aimed to map core concepts of a phenomenon or topic [14]. The procedure for this review followed the five stages set out in Arksey and O'Malley's [14] methodological framework for scoping studies, including: (1) identifying the research questions; (2) identifying relevant studies; (3) selecting studies; (4) charting the data; and (5) collating/summarizing the findings. The review was reported according to the Preferred Reporting Items for Systematic Reviews and Meta‐Analyses Extension for Scoping Reviews (PRISMA‐ScR) guidelines [15].

### 2.3 Search methods

An extensive literature search was conducted to identify published research articles and relevant grey literature (e.g., theses, research reports, and news articles) published/released between 2000 and 2022, with the goal of including only up‐to‐date evidence due to the significant growth of LGBT rights movements since the late twentieth century [16]. We searched eight commonly used electronic databases for published research articles: Embase, Scopus, ProQuest, PubMed, PsycINFO, PsychiatryOnline, CINAHL, and the Cochrane Library. The search terms used were: [(allyship) OR (all*)] AND [(work) OR (workplace*) OR (career*) OR (colleague*)] AND [(LGBT) OR (LGB) OR (lesbian) OR (gay) OR (bisexual) OR (transgender)] AND [(mental health) OR (psychological ill*) OR (mental disease*) OR (mental ill*) OR (psychological distress*)]. In addition, we searched three additional electronic databases for unpublished or grey literature and research theses/dissertations: Google Scholar, ProQuest Dissertations and Theses Global, and EthOS‐Dissertation. Additional literature was identified by screening the reference lists of the included articles. To maximize the search results, we also searched and identified relevant forums, or scholarly discussions/reports.

All database searches were conducted between January 1 and April 1, 2022. Covidence review manager was used for screening and reviewing identified studies. After removing duplications, two researchers (L. L. K. H. and A. T. C.) independently screened the titles and abstracts of all articles retrieved from the databases. The full texts of potentially eligible articles were then independently reviewed by two researchers (L. L. K. H. and C. C. Y. C.). In addition, the two researchers independently screened the reference lists of the reviews included in this study to avoid duplication of primary studies among the reviews.

### 2.4 Criteria for inclusion/exclusion

We included published articles reporting studies of any design and grey literature from all possible workplace contexts/cultures to identify available evidence addressing our research question. We excluded studies published in languages other than English and those without available full text.

### 2.5 Search outcomes

**Figure 1:**
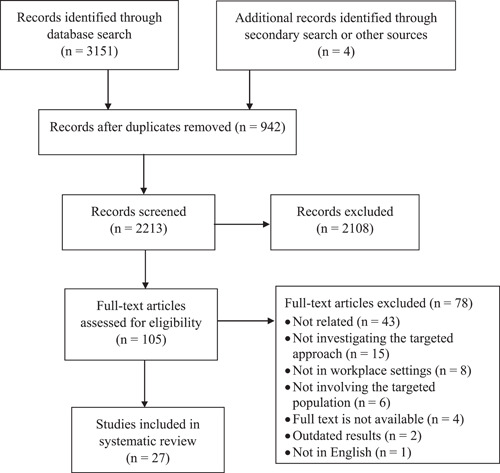
Flowchart of literature searching based on PRISMA guidelines.

Figure 1 shows the PRISMA flow diagram for this review. In total, 3155 articles were identified from the 11 electronic databases, a secondary search, and other sources, of which 942 were eliminated as duplicates. The titles and abstracts of the remaining 2213 articles were reviewed, and 105 articles underwent full‐text screening. Of these, 78 articles were excluded. Reasons for exclusion were that the articles were not: relevant to the research question (*n* = 43), studying workplace allyship (*n* = 15), conducted in workplace settings (*n* = 8), or involving LGBT people (*n* = 6). In addition, four articles were excluded because no full text was available and two because the results were outdated. Finally, 27 articles were included in this review. A data charting form was developed by the research team. Two researchers (L. L. K. H. and C. C. Y. C.) independently charted the data and then compared the results. Regular research team meetings were held to enhance the agreement and accuracy of the data analysis and interpretation and any disagreements/conflicts on the data extraction were resolved during the meeting.

## 3 RESULTS

### 3.1 Characteristics of the evidence and study samples

Table 1 presents a summary of the characteristics of all included articles. Of the 27 articles included in this review, most of them were published from 2011 to 2022, reported cross‐sectional studies, and were conducted in the United States (Table 2). The sample sizes in the reviewed studies ranged from 13 to 31,277 participants (median = 118), with most having 90–450 participants. The participants in the included studies were mostly recruited through online and offline advertisements (e.g., via pubs, bars, or local LGBT organizations). Participants' occupations were described in nine articles [17, 18, 19, 20, 21, 22, 23, 24, 25] but not reported/applicable in the remaining 17 articles. There was a range of occupational contexts from self‐employed or nonmanagement to industrial/executive management or professional (e.g., physician/lawyer) level.

**Table 1:** Characteristics of the included articles (*N* = 27).

Study and country	Study design/publication type	Population description	Sampling method	Occupational characteristics
Allan et al. [26], United States	Cross‐sectional study	LGB employees	Convenience sampling (online)	Unspecified
Arif et al. [27], Canada	Narrative review (grey literature, commentary article)	N/A	N/A	Unspecified
Boyles [17], United States	Cross‐sectional study (grey literature, dissertation)	LGB employees	Convenience sampling (online)	From nonmanagement to executive management level
Brooks and Edwards [25], United States	Qualitative study	LGBT employees and non‐LGBT allies	Purposive sampling	Employees of small companies or from educational settings
Chakrapani et al. [18], India	Qualitative study	Transmasculine people	Purposive sampling (maximum diversity sampling)	Employees of private companies or self‐employed
Couser [28], United States	Literature review	N/A	N/A	N/A
das Nair and Fairbank [12], United Kingdom	Narrative review (book chapter)	LGB people	N/A	N/A
Dupreelle et al. [41], United States	Cross‐sectional study (grey literature, report)	LGBTQ employees and non‐LGBTQ employees	National survey	Unspecified
Flanders et al. [19], United States	Qualitative study	Bisexual and non‐monosexual employees	Convenience sampling (fliers in public areas)	Business and university classes
Gates et al. [20], Australia	Cross‐sectional study	LGBTQ+ employees and other employees	Convenience sampling	From frontline service to senior leadership level
Goldberg & Smith [29], United States	Cohort study	Same‐sex couples	Convenience sampling	Unspecified
Hastings et al. [30], United States	Qualitative study	LGBTQ employees	Snowball sampling	Unspecified
Holman et al. [31], United States	Cross‐sectional study	LGBQ employees	Snowball and purposive sampling	Unspecified
Huffman et al. [32], United States	Cross‐sectional study	LGB employees	Convenience sampling (patrons at gay‐supportive places)	Unspecified
Luhtanen [33], United States	Cross‐sectional study	LGB people in the greater Buffalo area	Convenience sampling (survey)	Unspecified
Masuwa and Monica [34], United Kingdom	Presentation slides (grey literature)	N/A	N/A	N/A
Mara et al. [35], N/A	Systematic review	N/A	N/A	N/A
Miner and Costa [21], United States	Cross‐sectional study	LGB employees and non‐LGBTQ employees	Convenience sampling (online survey)	Restaurant employees
Perales [22], Australia	Cross‐sectional study	LGBTQ+ employees	National survey	Unspecified
Perales et al. [36], Australia	Cross‐sectional study	Transgender employees	National survey	Unspecified
Roland et al. [37], United States	Narrative reviews (grey literature, commentary article)	N/A	N/A	N/A
Ruggs et al. [23], United States	Cross‐sectional study	Transgender employees	Convenience sampling (from a larger study among transgender employees)	Professionals (e.g., lawyers, physicians), education, retail, and accommodation/food service or other industries
Seiler‐Ramadas et al. [38], Austria, Croatia, Serbia, Slovakia, Spain, and the United Kingdom	Qualitative study	LGBT employees	Snowball and chain sampling strategies	Unspecified
Singh and O'Brien [24], United States	Cross‐sectional study	LGBTQ employees	Convenience sampling (online survey)	Wide range of occupations
Smith & Ingram [39], United States	Cross‐sectional study	LGB employees	Convenience and snowball sampling	Unspecified
Griffiths [40], United Kingdom	News article (grey literature)	N/A	N/A	N/A
Webster et al. [13], United States	Systematic review with meta‐analysis	N/A	N/A	N/A

Abbreviation: LGBTQ, lesbian, gay, bisexual, transgender, and queer.

**Table 2:** Characteristics of the evidence and study samples.

Characteristics of the evidence (*N* = 27)	*N* (%)
Year of publication	
2011–2022	20 (74.1)
2002–2009	6 (22.2)
Did not report	1 (3.7)
Types of articles	
Journal articles or book chapters	21 (77.8)
Grey literature articles	6 (22.2)
Study design	
Cross‐sectional	13 (48.1)
Qualitative	5 (18.5)
Narrative review	3 (11.1)
Literature review	1 (3.7)
Systematic review	1 (3.7)
Systematic review and meta‐analysis	1 (3.7)
Prospective cohort study	1 (3.7)
Others	
News article	1 (3.7)
Presentation slides	1 (3.7)
Study country	
United States	17 (63.0)
United Kingdom	3 (11.1)
Canada	1 (3.7)
Australia	3 (11.1)
India	1 (3.7)
More than one country	1 (3.7)
Did not report	1 (3.7)
Characteristics of the study samples (*N* = 20)	
LGB/LGBT/LGBTQ employees	16 (80.0)
Transgender employees only	3 (15.0)
Bisexual employees only	1 (5.0)

Abbreviation: LGBTQ, lesbian, gay, bisexual, transgender, and queer.

### 3.2 Effects of workplace allies on psychological distress

#### 3.2.1 Supportive workplace relationships and climates

Table 3 summarizes the relevant findings of the included studies. Given that a workplace allyship could enhance supportive workplace relationships and climates, we found eight articles that considered the effects of this function on psychological distress among LGBT employees. A systematic review with a meta‐analysis of 27 correlational studies found the highest average corrected meta‐analytic (negative) correlation was between LGBT employees' psychological distress and supportive workplace relationships (*r* = –0.32), followed by a supportive workplace climate (*r* = –0.29) [13]. However, that study did not clearly state the types of workplace relationships (e.g., coworker or subordinate‐supervisor relationships), and did not conduct subgroup analyses of different groups within the LGBT community. Another cross‐sectional study found that a supportive workplace climate was positively associated with life satisfaction among LGB employees (*r* = –0.27, *p* < 0.01) [26]. A literature review stated that a supportive workplace climate and supportive relationships (particularly with coworkers) could be protective factors against workplace psychological distress among minority groups, such as the LGBT community [28]. Conversely, an unsupportive workplace climate, including experiences of workplace hetero‐sexism and unsupportive social interactions (e.g., expressions of disdain toward LGBT employees and unfavorable attention/comments) was associated with a higher level of psychological distress (mainly depression) among LGB [30, 39] and LGBT employees [21, 24, 38].

**Table 3:** Summary of the relevant findings of the included studies (*N* = 27).

Study	Relevant findings
Allan et al. [26]	A supportive LGB workplace climate was positively associated with life satisfaction, with job satisfaction having a mediating effect.
Arif et al. [27]	Four tools for developing allyship: (1) self‐education (educate oneself about others’ identities/experiences); (2) self‐challenge (challenge own discomfort and prejudices); (3) dedication in time and patience (dedicate the time/patience to learning how to be an ally); and (4) action (take action to promote change toward personal, institutional, and societal justice/equality).
Boyles [17]	Positive interactions with co‐workers and a perceived psychologically safe workplace environment could reduce psychological distress regarding identity disclosure and improve workplace engagement for LGB employees.
Brooks and Edwards [25]	LGBT employees considered the presence of workplace allies as vital and valuable, and fulfilling their primary needs for inclusion, safety, and equity.
	Non‐LGBT employees were motivated to be allies by witnessing injustices to their LGBT co‐workers. They provided interpersonal support for and confronted discrimination against LGBT co‐workers.
	Human resources department professionals could play an important role in serving as LGBT allies.
Chakrapani et al. [18]	Supportive relationship with co‐workers was considered a crucial factor for reducing the psychological distress of transgender employees.
	Workplace discrimination experiences could increase psychological distress.
	These experiences varied depending on transgender employees' disclosure of gender identity.
Couser [28]	Supportive workplace environment and network systems for minority groups (e.g., sexual minority groups) could be protective factors against workplace psychological distress (particularly depression).
	Co‐workers played an important role in developing a supportive culture that emphasized fairness and respected others' values/beliefs.
das Nair and Fairbank [12]	Discrimination against sexual minority groups could cause or worsen psychological distress.
	Unsupportive workplace environment could engender feelings of isolation/rejection, causing LGB people to conceal their LGB status from others (e.g., co‐workers), which resulted in an increased level of psychological stress.
Dupreelle et al. [41]	Compared with those who did not participate in workplace allyship programs, non‐LGBTQ employees who participated in these programs were two times more likely to recognize discrimination and three times more likely to intervene in discrimination events against LGBT employees.
	Compared with those who worked at companies without an ally group, non‐LGBTQ employees who worked at companies with an ally group (including those who did not join the ally group) were more likely to recognize discrimination and take action.
Flanders et al. [19]	Institutional support (e.g., employers supporting bisexual/nonmonosexual employees openly or celebrating Bisexuality Day events at the company) could have positive effects on the psychological well‐being and working experiences of bisexual/non‐monosexual employees.
Gates et al. [20]	Employees who had a greater alignment between personal and organizational values were more likely to be allies for co‐workers from minority groups (e.g., sexual minority groups), and showed greater commitment to organizational social justice and inclusion.
Goldberg and Smith [29]	Higher perceived workplace support was associated with lower depression and anxiety.
Hastings et al. [30]	There were negative impacts on the psychological health of LGBTQ employees when they chose to conceal their LGBTQ status, resulting from (1) disdain/unfavorable attention and comments expressed by co‐workers toward those who came out, (2) coworkers' rejection of those who came out, and (3) disclosing a person's LGBTQ status without their consent.
	Recommendations for enhancing allyship were: (1) cultivating a non‐face‐threatening climate, (2) providing visual and vocal support from allies, and (3) acknowledging the world beyond the organization.
Holman et al. [31]	Four work climates were identified and categorized: (1) supportive, (2) tolerant, (3) ambiguous, and (4) hostile.
	A supportive work climate facilitated the implementation of organizational policy/practice to prevent discrimination and promote inclusion/support for LGBT employees.
Huffman et al. [32]	Workplace support for LGB employees could be identified at several levels including support from co‐workers, supervisors, and organizations.
	Coworker support was positively associated with life satisfaction.
	Supervisor support was positively associated with job satisfaction.
	Organizational support was positively associated with disclosure of LGB status.
Luhtanen [33]	Acceptance/support from co‐workers was positively associated with the level of self‐esteem and negatively associated with the level of depression among gay/bisexual men.
Masuwa and Monica [34]	An LGBT ally was defined as “any person who supports and stands up for the rights of the LGBT community.”
	Allies can be any co‐workers including non‐LGBT employees.
	Allies work to improve the work climate, involve in movements for change and make a significant contribution to LGBT rights movements.
Mara et al. [35]	Several coping strategies for workplace discrimination at the individual level toward LGBT employees were identified and summarized: (1) internal, do not tend to seek help and conceal their LGBT status; (2) external, coping with the discrimination outside the workplace (e.g., health/legal services); (3) reactive, take defensive actions only when they sure they would not lose their job (e.g., complain to the human resources department); and (4) proactive, form allyship with co‐workers (including non‐LGBT coworkers).
	Organizational strategies for coping with workplace discrimination toward LGBT employees were: (1) creating a diverse/inclusive/safe work environment; (2) raising awareness and promoting education on LGBT issues; (3) forming LGBT employees' support groups (i.e., allyship for LGBT employees); (4) advancing employment policies; and (5) enforcing nondiscriminatory policies.
Miner and Costa [21]	Experiences of workplace hetero‐sexism among sexual minority employees were positively associated with the level of psychological distress, feelings of fear/anger, and physical health complaints, and negatively associated with job satisfaction.
Perales [22]	Level of workplace well‐being of LGBTQ+ employees was positively associated with the proportion of coworkers who considered them as allies, employees who received ally training, and organizational support in workplace allyship.
	There were statistically significant differences in workplace well‐being across LGBT subgroups, with “female, cis‐gender, and nonheterosexual” (lesbian/bisexual women) and “male, cis‐gender, nonheterosexual” (gay/bisexual men) reporting higher levels of well‐being than their other LGBTQ+ counterparts.
	Nonbinary employees reported lower levels of workplace well‐being when compared to LGB employees.
Perales et al. [36]	The use of inclusive language at work was associated with a higher level of workplace well‐being among transgender employees.
Roland et al. [37]	Six essential attitudes to becoming an effective ally: (1) always be humble, (2) be comfortable understanding own capacity of allyship, (3) be dedicated to learning, (4) be representative, (5) recognize own position and listen to the community, and (6) understand interconnections between groups.
Ruggs et al. [23]	Co‐workers’ reaction to the transgender identity was the most important factor for lowering perceptions of discrimination among transgender employees.
	Organizational policies also minimized the level of perceived discrimination.
	Disclosure of a transgender identity was not associated with the level of perception of discrimination.
Seiler‐Ramadas et al. [38]	Workplace discrimination could have negative impacts on LGBT employees.
	Unsupportive workplace climate was associated with a higher level of psychological distress among LGBT employees.
	Strategies are needed to ensure workplace diversity and equality.
Singh and O'Brien [24]	Work stress caused by coworkers' incivility and unsupportive workplace environment for LGBT employees was positively associated with psychological distress, internalized discrimination/shame (i.e., negative attitudes toward oneself), and levels of burnout.
	Increased psychological flexibility, which referred to acceptance, mindfulness, and cognitive fusion, could be a useful method for LGBT employees to respond to negative workplace experiences.
Smith and Ingram [39]	Experiences of workplace hetero‐sexism and unsupportive social interactions were positively associated with depression and psychological distress among LGB employees.
Griffiths [40]	Effective allyship included:
	Offering mental health first aid training for staff to act as workplace allies.
	Coordinating events aiming to remove the stigma of LGBT.
	Holding a seminar to educate workers to support LGBT employees.
	Helping to launch platforms that support LGBT workers/organizations.
Webster et al. [13]	Three workplace supports were identified: (1) LGBT policies and practices, (2) LGBT supportive climate, and (3) supportive workplace relationships.
	Supportive workplace relationships were more strongly related to work attitudes and psychological well‐being (e.g., work strain/stress) than the other two workplace supports.
	LGBT‐supportive climate was more strongly related to disclosure of LGBT status and perceived discrimination than the other two workplace supports.

Abbreviation: LGBTQ, lesbian, gay, bisexual, transgender, and queer.

#### 3.2.2 Disclosure of their sexual orientation or gender identity

The workplace environment/climate influenced decisions among LGBT employees regarding disclosing their sexual orientation or gender identity to their supervisors and coworkers. This was an important issue that greatly affected the psychological health of LGBT employees [6]. The aforementioned systematic review and meta‐analysis found the highest average corrected meta‐analytic correlation (positive) was between disclosure and a supportive workplace climate (*r* = 0.56), followed by supportive workplace relationships (*r* = 0.32) and organizational policies/practices (*r* = 0.29) [13]. A cross‐sectional study reported the perception of a safe workplace was negatively associated with psychological distress concerning disclosure (unstandardized coefficient *b* = –0.40, *p* < 0.000) and positively associated with a sense of inclusion/engagement (unstandardized coefficient *b* = 0.24, *p* < 0.000) [17]. However, in a workplace that was perceived to be unsafe, identity management strategies were used by LGBT employees to conceal their sexual orientation and prevent discrimination and harassment. Such strategies may result in a sense of social isolation and therefore a high level of psychological distress [12, 30]. A qualitative study also reported that LGBT employees' psychological well‐being was greatly affected when coworkers violated their trust by disclosing their LGBT status without their consent [30].

#### 3.2.3 Different levels of workplace ally support

A cohort study found that workplace support was negatively associated with depression (model coefficient *γ* = –2.06, *p* < 0.05) and anxiety (model coefficient *γ* = –2.31, *p* < 0.05) in LGBT employees. However, that study did not clearly describe the type of support [29]. Six articles reported that support from different levels of a company may have different positive psychological outcomes among LGBT employees. Three cross‐sectional studies found that support from coworkers was negatively associated with psychological distress and positively associated with life satisfaction among LGBT employees [17, 32, 33], which was consistent with the findings of a qualitative study [18]. A cross‐sectional study involving 99 LGB employees found that support from supervisors was positively associated with the level of job satisfaction (*r* = 0.52, *p* < 0.01) and an LGB‐supportive working climate (*r* = 0.36, *p* < 0.01) [32]. A national‐based survey of 31,277 employees (including 5528 LGBTQ+) in Australia found that the level of workplace well‐being of LGBTQ+ employees was positively associated with the proportion of co‐workers who considered them as allies (*β* = 15.98, *p* < 0.01), employees who received ally training (*β* = 9.82, *p* < 0.01), and organizational support in workplace allyship (*β* = 9.84, *p* < 0.01) [22]. The survey also showed that nonbinary employees had significantly lower levels of workplace well‐being than LGB employees, implying that more diversity training was needed for transgender/nonbinary employees [22]. In addition, a qualitative study with 91 LGB individuals suggested that organizational support had positive effects on their psychological well‐being [19].

#### 3.2.4 Workplace discrimination

Workplace allyship was suggested to be a potentially effective approach to reduce workplace discrimination. However, the effectiveness of allyship in reducing workplace discrimination was not evaluated, and most of the suggested strategies merely laid a theoretical basis for future research. Workplace discrimination of minority groups such as LGBT employees may cause or worsen psychological distress [12, 38]. A qualitative study involving 27 transgender employees found experiences of workplace discrimination were closely related to high levels of psychological stress [18]. In a cross‐sectional study targeting transgender employees, the level of perceived discrimination against these employees was significantly associated with supportive coworker relationships (*β* = –0.49, *p* < 0.001) and organizational policies (*β* = –0.15, *p* < 0.05), but not with disclosure of their gender identity (*β* = –0.09, *p* > 0.05) [23]. A systematic review of 52 studies (narrative reviews/case studies) identified four strategies (i.e., internal, external, reactive, and proactive strategies) that LGBT employees and organizations used to cope with workplace discrimination against LGBT employees [35].

### 3.3 Essential or effective structure/components of workplace allyship

We identified six articles clearly defined an ally as a member of the majority group in the workplace (e.g., non‐LGBT people), who aimed to prevent the oppression of a minority group (e.g., LGBT people), support them, and advocate for their rights [13, 20, 25, 27, 34, 37]. Workplace allies performed their functions by enhancing supportive workplace relationships and climates, and creating diverse, inclusive, and safe workplaces for coworkers who were members of the minority group [17, 26, 28, 31, 38, 41]. A supportive and safe workplace may be considered a more comfortable environment for LGBT employees to either disclose their LGBT status or discuss LGBT‐related issues [25, 41]. A qualitative study from the US that interviewed eight LGBT employees indicated that workplace allies (non‐LGBT coworkers) helped them fulfill their primary needs of equity, inclusion, and safety [25]. A report (grey literature) of a national‐based survey in the United States emphasized the importance of an allyship program as an effective method of fostering the inclusion of LGBT employees by educating and engaging non‐LGBT coworkers. Moreover, non‐LGBT coworkers who had participated in workplace allyship programs were two to three times more likely to recognize discrimination and intervene in discrimination events against LGBT coworkers than nonparticipants [41]. Addressing intersectionality in allyship programs, incorporating diversity and inclusion in managerial performance assessments, designating a confidential ombudsman to monitor ally programs, and establishing pronoun guidelines were considered essential components of an effective allyship program [20, 41]. It is important to note that workplace allies are more comfortable, confident, and willing to speak up for LGBT employees when they feel that this aligns with their organizational values and trust that the organization will support their actions for social justice [20].

Six articles mentioned the essential elements of allyship or made recommendations regarding developing allyship. In summary, we identified three positive and essential components of workplace allyship for LGBT employees: knowledge, empathy, and action.

#### 3.3.1 Knowledge

Self‐motivated learning about correct information regarding LGBT was considered an important step to understand the experiences of LGBT employees and recognize the oppression they faced [27]. Knowledge regarding LGBT (e.g., use of inclusive languages) could be gained from conversations between LGBT employees and searching related information from several different sources (e.g., the Internet or social media) [27, 36]. In addition, workplace allies with adequate knowledge about LGBT could enact changes in attitudes/behaviors among themselves and non‐LGBT coworkers toward misconceptions/stereotypes of LGBT issues [25]. This contributed to creating an inclusive and supportive workplace climate to promote mental well‐being among LGBT employees Couser [28].

#### 3.3.2 Empathy

Workplace allies could provide interpersonal support for LGBT colleagues through offering acceptance and friendship and including them in social interactions [25]. Allies could self‐reflect on their motivation and behaviors from time to time so as to prevent personal bias toward LGBT employees [27].

#### 3.3.3 Action

**Figure 2:**
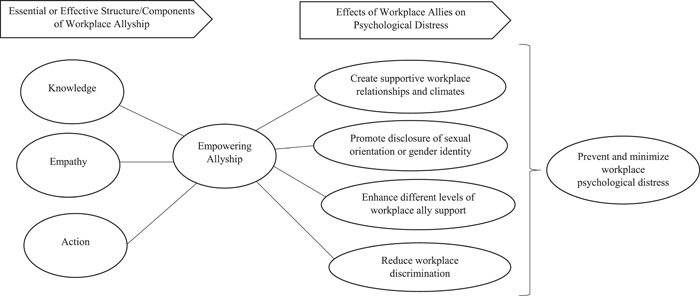
A diagram for illustrating the main findings.

Workplace allies for LGBT employees could take actions to prevent/eliminate discrimination/harassment and promote genuine equity [25, 27, 34]. Allies stood up for and defended the LGBT employees when discrimination/harassment was witnessed, and amplified the suppressed voices to ensure ideas from these employees were heard and efforts given credit [27]. Workplace allies may play an advocator role to report confronting discrimination through social action, subtle organizational maneuvering, and speaking out against prejudiced language/behaviors toward LGBT employees [25]. Figure 2 depicts the main findings of this review.

### 3.4 Recommendations for research, practice, and policy

As shown in the literature, allyship refers to a majority or dominant group(s) that supports a minority group [13, 20, 22, 25, 27, 34, 37]. In this section, we expand the concept of allies to go beyond the individual level and include entities (e.g., organizations).

(1) *Recommendations for further research*
  There is little empirical evidence and identified grey literature in countries beyond the United States, given that 68% of the included studies were conducted in the United States. This underscores the importance of understanding the situations, needs, and psychological well‐being of LGBT employees around the world, especially in the context of traditional cultural and societal norms (e.g., the Chinese community). In addition, more studies focused on LGB employees than on transgender employees (LGB vs. Transgender: nine vs. three studies), which indicated that evidence regarding transgender employees remains scarce and suggested further efforts are needed to explore issues related to transgender employees. Additional attention and research (e.g., qualitative studies) are also warranted to further understand the differences in workplace allyship needs and opinions in diverse LGBT communities, as well as among LGB versus transgender people.
  Moreover, the included studies exhibited high levels of heterogeneity in their target samples, with diverse occupational contexts (from self‐employed to nonmanagement to industrial/executive management or professional levels). It may be that LGBT employees in different workplace settings may have different workplace experiences and subsequently experience different psychological distress. Further research could focus on individual workplace settings and compare the levels of psychological distress experienced by LGBT employees across workplace contexts.
  Most of the included studies used cross‐sectional or qualitative designs, which might have restricted the identification of temporal/causal relationships between workplace allyship and psychological distress, as well as other contributing factors. The effectiveness of workplace allyship in addressing or preventing psychological distress could be best explored through randomized controlled trials or longitudinal studies, which would increase the quality of evidence regarding possible causal inferences or long‐term impacts of workplace allyship. Some of the articles included in this review outlined criteria for guiding people to establish allyship for LGBT employees. However, the development of these guidelines did not have adequate literature support and was not based on recent best‐available evidence. Further research is needed to validate existing guidelines for establishing workplace allyship for LGBT employees using an evidence‐based approach to enhance the quality of recommendations on the development of LGBT workplace allyship.
(2) *Recommendations for practice*
  Based on the findings of this review, the following are recommendations codeveloped by our research team and a group of stakeholders, including LGBT employees and leaders/founders of LGBT organizations. These recommendations following the scoping review would be useful as a starting point for companies interested in creating an inclusive work environment for LGBT employees by developing/promoting allyship programs within their workplaces.
  It was not only coworkers and supervisors but also work teams and staff in a department or even the entire company could serve as workplace allies for LGBT employees [19, 25, 32, 35]. Based on the three‐step model of building inclusive workplace culture [41] and the LGBT Climate Inventory of four workplace climates (i.e., supportive, tolerant, ambiguous, and hostile) [31] identified in this review, we developed steps that could be implemented by small‐ and medium‐sized enterprises at different stages in adjusting the work climate related to LGBT issues to strengthen workplace allyship (Table 4). We targeted small‐ and medium‐sized enterprises for two main reasons: (1) organizational culture varies based on the company size [21]; and (2) most existing diversity and inclusion programs tend to target large‐sized businesses and industries (e.g., multinational corporations and the finance and banking industry) but rarely reach out to small‐ and medium‐sized enterprises. The suggested stages and the corresponding measures shown in Table 4 would provide initial step‐by‐step guidance on how small‐ and medium‐sized enterprises could create a more LGBT‐friendly workplace environment by promoting workplace allyship [35].
  We considered the four workplace climates (i.e., supportive, tolerant, ambiguous, and hostile) [31] as representing distinct stages of inclusiveness and offered specific recommendations to develop workplace allyship and improve inclusivity in response to each stage (Table 4). According to Holman et al. [31], a supportive workplace climate indicates a low likelihood of workplace discrimination and a high likelihood of reporting workplace support; a tolerant workplace climate indicates a relatively high level of workplace support, but coworkers may feel less comfortable discussing LGBT‐related humor and LGBT employees' relationships; an ambiguous workplace climate indicates relatively similar high levels of workplace discrimination and support; a hostile workplace climate indicates to a greater likelihood of discrimination than support, making LGBT employees feel pressured to conceal their sexual orientation or gender identity. Companies could be encouraged to assess their specific workplace climate such as using the LGBT Climate Inventory [31], which would provide an index for a department/company to position itself in developing an allyship program according to its LGBT issues‐related work climate stage (i.e., from hostile to supportive). In addition to making general recommendations for all levels of management involved, we also tailored suggested measures for particular levels of management that we believe contribute/exert more effort to improve inclusivity. Between different stages of establishing a supportive work climate related to LGBT issues (Table 4), evaluation should be conducted to monitor progress and plan for further action [20, 41].
  In addition to the training of non‐LGBT and LGBT allies within companies (internal measures), the concept of allyship can be expanded to include promoting an overall LGBT‐inclusive business environment (external measures); in other words, building allies between businesses with different sizes. A network of good practices and idea exchange on LGBT allyship among businesses might foster a supportive macroworkplace environment for LGBT employees. For example, such a network can provide an open platform for businesses to share and learn more about good allyship practices or collaborate with local LGBT initiatives and academic institutions to provide resources for evidence‐based research to minimize or prevent workplace psychological distress among LGBT employees [40].
(3) *Recommendations for policy*
  A number of articles highlighted the importance of identifying the types of support provided (e.g., supervisor or coworker support) to successfully run an allyship program in an organization [32, 35]. These findings suggested that allyship at the workplace should be built using both top‐down and bottom‐up processes, as good organizational policies could encourage employees' active participation, and employees' enthusiasm may cultivate a better workplace climate for LGBT employees [17, 19, 32, 33]. As such, organizations that are enthusiastic in building allyship for LGBT employees can be equally committed to connecting with other organizations to exchange policies and encourage other organizations (e.g., small businesses) to become involved. This may result in better development of workplace allyship(s) and nurture a more accepting macroenvironment for LGBT employees.

**Table 4:** Steps for different stages of work climate related to LGBT issues for developing workplace allyship.

Stage	Work climate	Level of management involved	Suggested measures	Purpose	Details and examples
1.	Hostile/ignorant	All	Regular education on LGBT issues (e.g., addressing religious issues)	Awareness and education	Consult and invite LGBT groups to share information on LGBT issues Putting up fact sheets/articles in public areas (e.g., pantries, resting areas, and washrooms) Consult and invite LGBT groups to provide training (or training materials) Role play on dealing with possible scenarios Address intersectionality Focus on day‐to‐day touches rather than formal procedures Encourage the use of inclusive language
		Managerial and above (particularly human resources department staff)	Basic LGBT diversity training	Awareness and education	
2.	Ambiguous	All, with a focus on co‐workers	Regular education on allyship as an approach to support LGBT	Awareness and education	Consult and invite LGBT groups to provide training (or at least training materials) Putting up fact sheets/articles in public areas (e.g., pantries, resting areas, and washrooms) Add survey questions related to LGBT issues into company surveys or Create an independent survey on LGBT issues Senior staff and managers serve as champions (e.g., how they can help resolve conflicts that arise over LGBT‐related issues among employees) Managerial staff serve as examples of good allies Specific protocols for the human resources team (e.g., confidentiality of employee records) Add inclusive facilities (e.g., gender‐neutral toilets) Use inclusive languages in company policies
		All	Stocktaking survey on LGBT population and employees' views about LGBT issues, such as willingness to be allies (specifically targeting non‐LGBT employees) or possible discrimination faced by LGBT employees	Stocktaking	
		Managerial	Mediation training	Resolving conflicts	
		Policy	Review existing policies	Inclusiveness	
3.	Tolerant	All	Introduction of the allyship program as part of the staff development program	Forming support network	Recruit allies/champions to target one‐third of a company to be allies Codesign allyship program based on company size, culture, and employee needs Consult LGBT groups to give advice on policy design Draw references from corporations with well‐developed diversity and inclusion programs and policies Seek advice from academics with relevant research background Seek legal advice
		Policy	An early stage of policy design addressing the discrimination faced by LGBT employees (e.g., human resources policies, employee benefits, antidiscrimination policies)	Inclusiveness	
4.	Supportive	All	Establishment of a good practice‐sharing platform within the company	Awareness and education	Updates in both online and paper formats (e.g., hardcopy in public areas such as pantries, resting areas, and washrooms, or electronic copy on computers) Set up LGBT policies according to company circumstances Prioritize items with higher urgency, depending on employees' needs LGBT‐friendly attitude as one of the indicators for job performance and promotion Increase the percentage with the level of management (e.g., 5% for general employees, 7%–10% for senior and managerial staff) Set up a discriminatory panel for discriminative behavior Penalize employees with discriminative behavior, with worst case termination of the contract Invite collaboration with LGBT groups and academic institutions for a more systematic evaluation of the program
		Policy	Introduction of LGBT‐friendly policies	Inclusiveness	
		Policy	Introduction of a reward system and non‐discriminatory policies	Inclusiveness	
		Policy	Regular review of allyship program (yearly)	Evaluation	

Abbreviation: LGBT, lesbian, gay, bisexual, and transgender.

## 4 CONCLUSION

This scoping review addressed an exploratory research question and mapped the core concepts of workplace allies or allyship for LGBT employees, and it identified available evidence about the effects of workplace allies/allyship on minimizing or preventing psychological distress and its components. The findings of this review can increase employers' and stakeholders' understanding of the criteria for developing allyship and its possible effects on promoting psychological health among LGBT employees, enhancing workplace harmony, and increasing LGBT employees' engagement and job satisfaction. The findings also suggest workplace allyship can enhance supportive workplace relationships and climates, and create diverse, inclusive, and safe workplaces for LGBT employees. This may reduce LGBT employees' workplace‐related psychological distress (e.g., due to discrimination and harassment in the workplace). Three positive/essential components of workplace allies/allyship for LGBT employees were identified: knowledge, empathy, and action. A negative association between the functions of workplace allies and psychological distress among LGBT employees was found in the included studies, which were all nonintervention, cross‐sectional, or qualitative studies. Randomized controlled trials or longitudinal studies may be needed to investigate the effectiveness and predictive factors of workplace allyship in diverse workplace settings and across countries over a long‐term follow‐up period. In addition, the findings of qualitative studies show that LGBT employees value the presence of workplace allies, which allows these employees to be comfortable disclosing their LGBT status and engenders a sense of inclusion. In‐depth qualitative studies with purposive samples are recommended to better understand the experiences and perceived benefits of workplace allyship across different LGBT communities and workplace settings.

## AUTHOR CONTRIBUTIONS

**Laurie Long Kwan Ho**: Conceptualization; Formal analysis; Funding acquisition; Methodology; Project administration; Supervision; Writing—original draft. **Ankie Tan Cheung**: Conceptualization; Formal analysis; Funding acquisition; Methodology; Writing—review & editing. **Carlo Chak Yiu Chan**: Formal analysis; Methodology; Writing—review & editing. **Eliza Lai Yi Wong**: Methodology; Writing—review & editing. **Wilson Wai San Tam**: Methodology; Writing—review & editing. **Wai Tong Chien**: Conceptualization; Funding acquisition; Methodology; Project administration; Supervision; Writing—review & editing.

## CONFLICT OF INTEREST STATEMENT

The authors declare no conflicts of interest.

## Data Availability

Data sharing is not applicable to this article as no new data were created or analyzed in this study.

## References

[1] Bachmann CL , Gooch B . LGBT in Britain—Work Report. Stonewall; 2018. https://www.stonewall.org.uk/lgbt-britain-work-report

[2] Bauermeister JA , Meanley S , Hickok A , Pingel E , VanHemert W , Loveluck J . Sexuality‐related work discrimination and its association with the health of sexual minority emerging and young adult men in the Detroit metro area. Sex Res Social Policy. 2014;11(1):1‐10. 10.1007/s13178-013-0139-0 24659928 PMC3960079

[3] Sears B , Mallory C , Flores AR , Conron KJ . LGBT People's Experiences of Workplace Discrimination and Harassment. UCLA; 2021. https://escholarship.org/uc/item/5s95g1pg

[4] Boudrias V , Trépanier S‐G , Salin D . A systematic review of research on the longitudinal consequences of workplace bullying and the mechanisms involved. Aggress Violent Behav. 2021;56:101508. 10.1016/j.avb.2020.101508

[5] Kates J , Ranji U , Beamesderfer A , Salganicoff A , Dawson L . *Health and Access to Care and Coverage for Lesbian, Gay, Bisexual, and Transgender Individuals in the U.S*. The Henry J. Kaiser Family Foundation Headquarters. 2018. https://npin.cdc.gov/publication/health-and-access-care-and-coverage-lesbian-gay-bisexual-and-transgender-individuals-us

[6] Newheiser A‐K , Barreto M , Tiemersma J . People like me don't belong here: identity concealment is associated with negative workplace experiences: people like me don't belong here. J Social Issues. 2017;73(2):341‐358. 10.1111/josi.12220

[7] Conron KJ , Goldberg SK . Adult LGBT Population in the United States. The Williams Institute, UCLA; 2022. https://williamsinstitute.law.ucla.edu/wp-content/uploads/LGBT-Adult-US-Pop-Jul-2020.pdf

[8] Sharfman A , Cobb P . Sexual Orientation, UK: 2019. Office for National Statistics; 2021. https://www.ons.gov.uk/peoplepopulationandcommunity/culturalidentity/sexuality/bulletins/sexualidentityuk/2019

[9] Sabat IE , Martinez LR , Wessel JL . Neo‐activism: engaging allies in modern workplace discrimination reduction. Industr Organizat Psychol. 2013;6(4):480‐485. 10.1111/iops.12089

[10] Evans NJ , Wall VA , eds. Beyond Tolerance: Gays, Lesbians, and Bisexuals on Campus. American College Personnel Association, 1991.

[11] Lloren A , Parini L . How LGBT‐supportive workplace policies shape the experience of lesbian, gay men, and bisexual employees. Sex Res Social Policy. 2017;14(3):289‐299. 10.1007/s13178-016-0253-x

[12] das Nair R , Fairbank S . Mental health. In: das Nair R , Butler C , eds. Intersectionality, Sexuality and Psychological Therapies. 1st ed. Wiley; 2012:185‐211. 10.1002/9781119967613.ch8

[13] Webster JR , Adams GA , Maranto CL , Sawyer K , Thoroughgood C . Workplace contextual supports for LGBT employees: a review, meta‐analysis, and agenda for future research. Hum Resour Manage. 2018;57(1):193‐210. 10.1002/hrm.21873

[14] Arksey H , O'Malley L . Scoping studies: towards a methodological framework. Int J Social Res Methodol. 2005;8(1):19‐32. 10.1080/1364557032000119616

[15] Tricco AC , Lillie E , Zarin W , et al. PRISMA extension for scoping reviews (PRISMA‐ScR): checklist and explanation. Ann Intern Med. 2018;169(7):467‐473. 10.7326/M18-0850 30178033

[16] Institute of Medicine . The Health Of Lesbian, Gay, Bisexual, and Transgender People: Building a Foundation for Better Understanding. National Academies Press; 2011. 22013611

[17] Boyles PA . *“Thank You for Letting me be Myself”: Exploring the Effects of Identity Management Strategies on Engagement Levels of Lesbian, Gay and Bisexual Employees*. Ph.D. Thesis. Virginia Polytechnic Institute and State University; 2008.

[18] Chakrapani V , Scheim AI , Newman PA , et al. Affirming and negotiating gender in family and social spaces: stigma, mental health and resilience among transmasculine people in India. Cult Health Sex. 2022;24:951‐967. 10.1080/13691058.2021.1901991 33847243 PMC7612960

[19] Flanders CE , Tarasoff LA , Legge MM , Robinson M , Gos G . Positive identity experiences of young bisexual and other nonmonosexual people: a qualitative inquiry. J Homosex. 2017;64(8):1014‐1032. 10.1080/00918369.2016.1236592 27797650

[20] Gates TG , Achia T , Petch J . Allyship, social justice values, and commitment at an Australian social service organization. J Soc Serv Res. 2021;47(6):796‐807. 10.1080/01488376.2021.1924341

[21] Miner KN , Costa PL . Ambient workplace heterosexism: implications for sexual minority and heterosexual employees. Stress Health. 2018;34(4):563‐572. 10.1002/smi.2817 29856117

[22] Perales F . Improving the wellbeing of LGBTQ+ employees: do workplace diversity training and ally networks make a difference? Prev Med. 2022;161:107113. 10.1016/j.ypmed.2022.107113 35718120

[23] Ruggs EN , Martinez LR , Hebl MR , Law CL . Workplace “trans”‐actions: how organizations, coworkers, and individual openness influence perceived gender identity discrimination. Psychol Sexual Orientat Gender Diversity. 2015;2(4):404‐412. 10.1037/sgd0000112

[24] Singh RS , O'Brien WH . The impact of work stress on sexual minority employees: could psychological flexibility be a helpful solution. Stress Health. 2020;36(1):59‐74. 10.1002/smi.2913 31755638

[25] Brooks AK , Edwards K . Allies in the workplace: including LGBT in HRD. Adv Develop Human Resources. 2009;11(1):136‐149. 10.1177/1523422308328500

[26] Allan BA , Tebbe EA , Duffy RD , Autin KL . Living a calling, life satisfaction, and workplace climate among a lesbian, gay, and bisexual population. Career Dev Q. 2015;63(4):306‐319. 10.1002/cdq.12030

[27] Arif S , Afolabi T , Mitrzyk BM , et al. Engaging in authentic allyship as part of our professional development. Am J Pharm Educ. 2022;86:8690. 10.5688/ajpe8690 34385173 PMC10159490

[28] Couser GP . Challenges and opportunities for preventing depression in the workplace: a review of the evidence supporting workplace factors and interventions. J Occup Environ Med. 2008;50(4):411‐427. 10.1097/JOM.0b013e318168efe2 18404014

[29] Goldberg AE , Smith JZ . Stigma, social context, and mental health: lesbian and gay couples across the transition to adoptive parenthood. J Couns Psychol. 2011;58(1):139‐150. 10.1037/a0021684 21171740 PMC3081377

[30] Hastings SO , Minei E , Warren S . Organizational practices leading to closeting: the interactional construction of ‘closets’. J Appl Commun Res. 2021;49(6):687‐704. 10.1080/00909882.2021.1937673

[31] Holman EG , Fish JN , Oswald RF , Goldberg A . Reconsidering the LGBT climate inventory: understanding support and hostility for LGBTQ employees in the workplace. Journal of Career Assessment. 2019;27(3):544‐559. 10.1177/1069072718788324 33967571 PMC8100868

[32] Huffman AH , Watrous‐Rodriguez KM , King EB . Supporting a diverse workforce: what type of support is most meaningful for lesbian and gay employees. Hum Resour Manage. 2008;47(2):237‐253. 10.1002/hrm.20210

[33] Luhtanen RK . Identity, stigma management, and well‐being: a comparison of lesbians/bisexual women and gay/bisexual men. J Lesbian Stud. 2002;7(1):85‐100. 10.1300/J155v07n01_06 24815716

[34] Masuwa P , Monica S . Simple Guide to Allyship—The National Health Service . https://midlands.leadershipacademy.nhs.uk/wp-content/uploads/sites/3/2020/12/Allyship-Toolkit_.pdf

[35] Mara L‐C , Ginieis M , Brunet‐Icart I . Strategies for coping with LGBT discrimination at work: a systematic literature review. Sex Res Social Policy. 2021;18(2):339‐354. 10.1007/s13178-020-00462-w

[36] Perales F , Ablaza C , Elkin N . Exposure to inclusive language and well‐being at work among transgender employees in Australia, 2020. Am J Public Health. 2022;112(3):482‐490. 10.2105/AJPH.2021.306602 35196034 PMC8887154

[37] Roland A , Pachipala K , Webb A , Morris M , Park Y , Park JW . Engaging in the process of allyship: LGBTQ+ community . 2020. 10.25611/R17Z-T359

[38] Seiler‐Ramadas R , Markovic L , Staras C , et al. “I Don't even want to come out”: the suppressed voices of our future and opening the lid on sexual and gender minority youth workplace discrimination in Europe: a qualitative study. Sex Res Social Policy J NSRC: SR & SP. 2022;19:1452‐1472. 10.1007/s13178-021-00644-0 PMC8481111 34608404

[39] Smith NG , Ingram KM . Workplace heterosexism and adjustment among lesbian, gay, and bisexual individuals: the role of unsupportive social interactions. J Couns Psychol. 2004;51(1):57‐67. 10.1037/0022-0167.51.1.57

[40] Griffiths T . *Stonewall Ranks K&L Gates Among Best UK Companies for LGBT Employees*. 2017. https://www.klgates.com/Stonewall-Ranks-KL-Gates-Among-Best-UK-Companies-for-LGBT-Employees-01-19-2017

[41] Dupreelle P , Novacek G , Lindquist J , Micon N , Pellas S , Testone G . A New LGBTQ Workforce Has Arrived—inclusive Cultures Must Follow. Boston Consulting Group; 2021. https://web-assets.bcg.com/img-src/BCG-A-New-LGBTQ-Workforce-Has-Arrived-Inclusive-Cultures-Must-Follow-Jun-2020_tcm9-251548.pdf

